# Giant plexiform fibromyxoma of the esophagus

**DOI:** 10.1007/s00508-026-02740-w

**Published:** 2026-04-21

**Authors:** Paul Swatek, Edin Ahmic, Iva Brcic, Iurii Mykoliuk, Anton Busau, Andrej Roj, Jörg Lindenmann

**Affiliations:** 1 Department of Surgery, Division of Thoracic and Hyperbaric Surgery, Medical University of Graz, Auenbruggerplatz 29, 8036 Graz, Austria; 2 Diagnostic and Research Institute of Pathology, Medical University of Graz, Graz, Austria

**Keywords:** Esophageal tumor, Digestive system tumors, Dysphagia, Esophageal surgery, Positron emission tomography

## Abstract

Plexiform fibromyxoma is a very rare gastrointestinal tumor, usually located in the stomach, predominantly in the antrum region. There are some cases reporting the tumor location in the small intestine, whereas originating from the esophagus has been described only twice up to now. This is the first report of a fast growing and symptomatic giant esophageal plexiform fibromyxoma requiring transthoracic esophagectomy and reconstruction by gastric pull-up.

A 73-year-old patient was admitted with the clinical symptoms of dysphagia, chest pain, fever and weight loss for at least 2 months. A computed tomography (CT) scan revealed a giant mediastinal tumor measuring 11 × 7 × 7 cm with subsequent compression of the esophagus, the trachea and the heart. Endoscopy showed an ulcerated esophagus with signs of necrosis and obstruction of the esophageal lumen. A 18F-fluorodeoxyglucose positron emission tomography (FDG-PET) scan detected a massive tracer uptake of this esophageal tumor suggesting malignancy.

After discussion in the interdisciplinary tumor board transthoracic esophagectomy and reconstruction by gastric pull-up were performed. The patient fully recovered and was discharged on the 12th postoperative day. Contrary to the intraoperative frozen section indicating an esophageal sarcoma the final histopathological examination confirmed a giant esophageal plexiform fibromyxoma.

## Introduction

Plexiform fibromyxoma is a very rare gastrointestinal tumor, usually located in the stomach, predominantly in the antrum region. Up to now only a few cases have been reported, with the location of the tumor in the antrum and the fundus of the stomach as well as in the duodenum and the esophagus. Common symptoms are dysphagia, hematemesis, nausea, anemia and weight loss. Complete surgical resection of the tumor has been established as the gold standard of treatment [1, 2].

To the best of our knowledge this is the first report of a fast growing and symptomatic giant esophageal plexiform fibromyxoma requiring transthoracic esophagectomy and reconstruction by orthotopic gastric pull-up.

## Case report

**Fig. 1:**
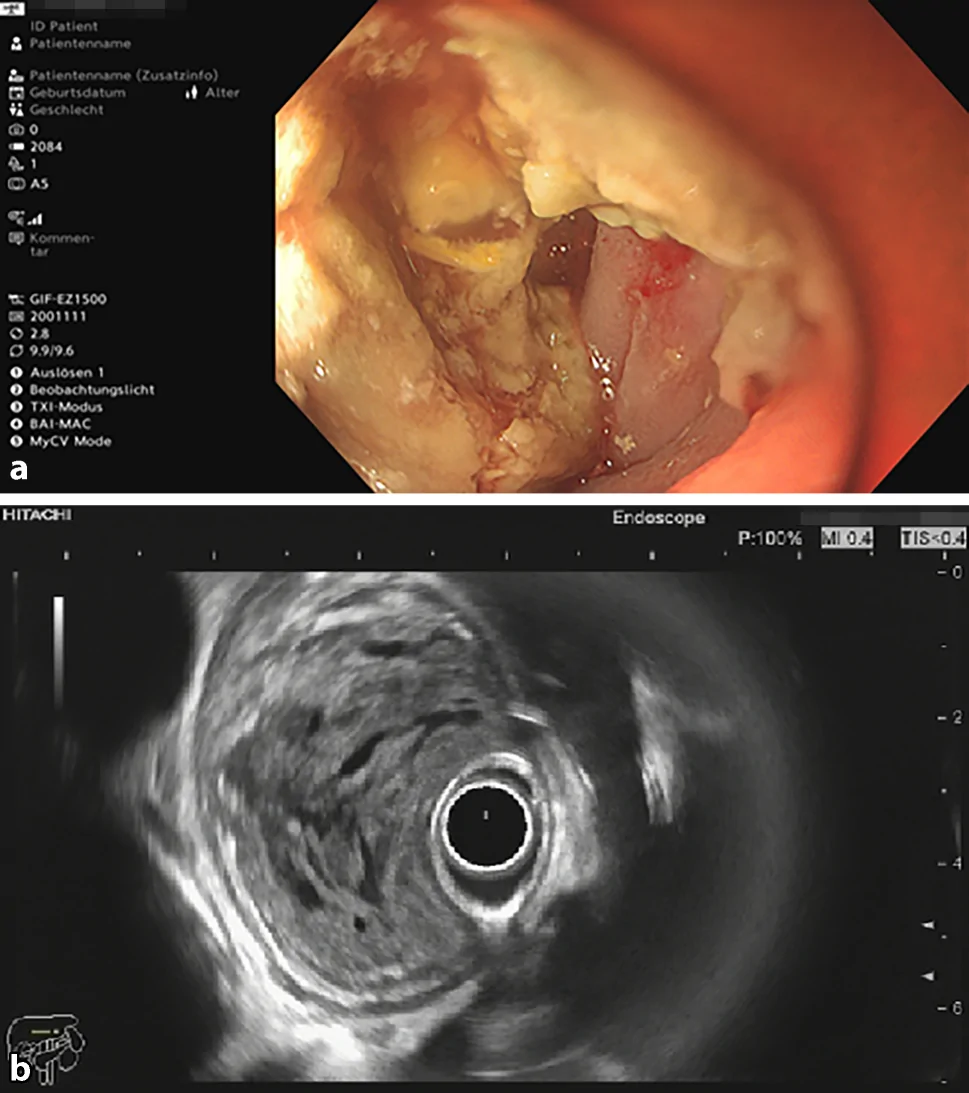
**a** Endoscopic view of the tumor showed necrosis of the surface, **b** endoscopic ultrasound (EUS) image showing suspected T3 growth of the tumor

A 73-year-old patient was admitted to hospital with symptoms of gastroesophageal reflux which did not respond to proton pump inhibitors (PPI), dysphagia, chest pain as well as fever and weight loss (5 kg in 2 months). Moreover, a considerable leucocytosis of 14.8310^9^/L and slightly elevated levels of C‑reactive protein 17.7mg/L were detected. Flexible esophagoscopy showed a subtotal stenosis of the middle to distal esophagus located between 29 and 35 cm from the incisors, with ulcerated esophageal mucosa and necrosis (shown in Fig. 1a). Repeated biopsies were taken from different sites of the tumor. The specimen was interpreted as nonmalignant tissue corresponding to highly inflammatory esophageal alterations. Endoscopic ultrasound (EUS) revealed tumor infiltration of the esophageal wall reaching the muscle layer corresponding to a T3 situation (shown in Fig. 1b). Endoscopic ultrasound-guided fine needle aspiration (EUS-FNA) as well as endobronchial ultrasound-guided transbronchial needle aspiration (EBUS-TBNA) were performed; however, neither procedure yielded representative diagnostic material. Flexible bronchoscopy confirmed slight extrinsic compression of both main bronchii due to the large volume of this giant esophageal tumor.

**Fig. 2:**
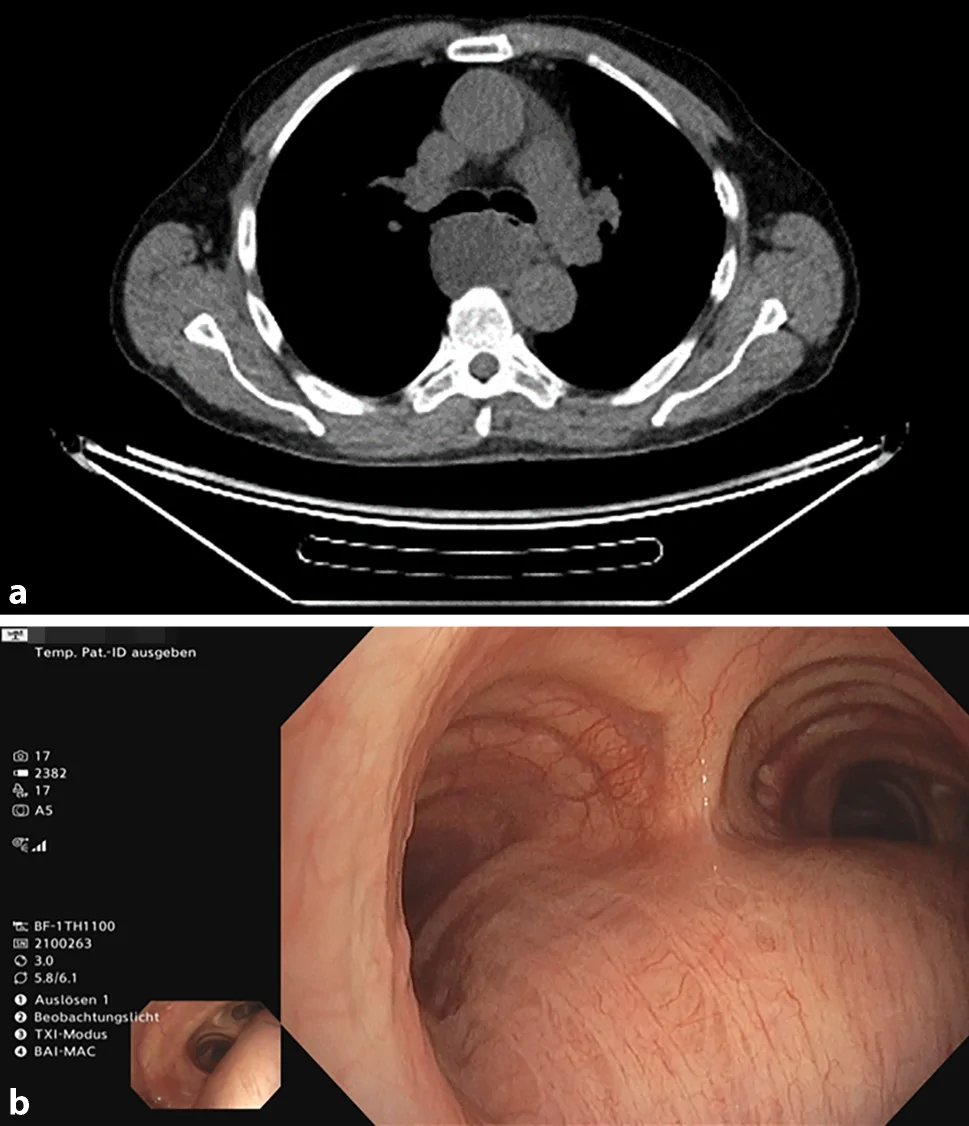
**a** Axial computed tomography (CT) image of the thorax showing the tumor compressing both main bronchi **b** corresponding endoscopic finding of dorsal compression of the main bronchi

The CT scan confirmed this giant tumor measuring 11 × 7 × 7cm located at the middle third of the esophagus with subsequent compression of both main bronchii (shown in Fig. 2a, b).

**Fig. 3:**
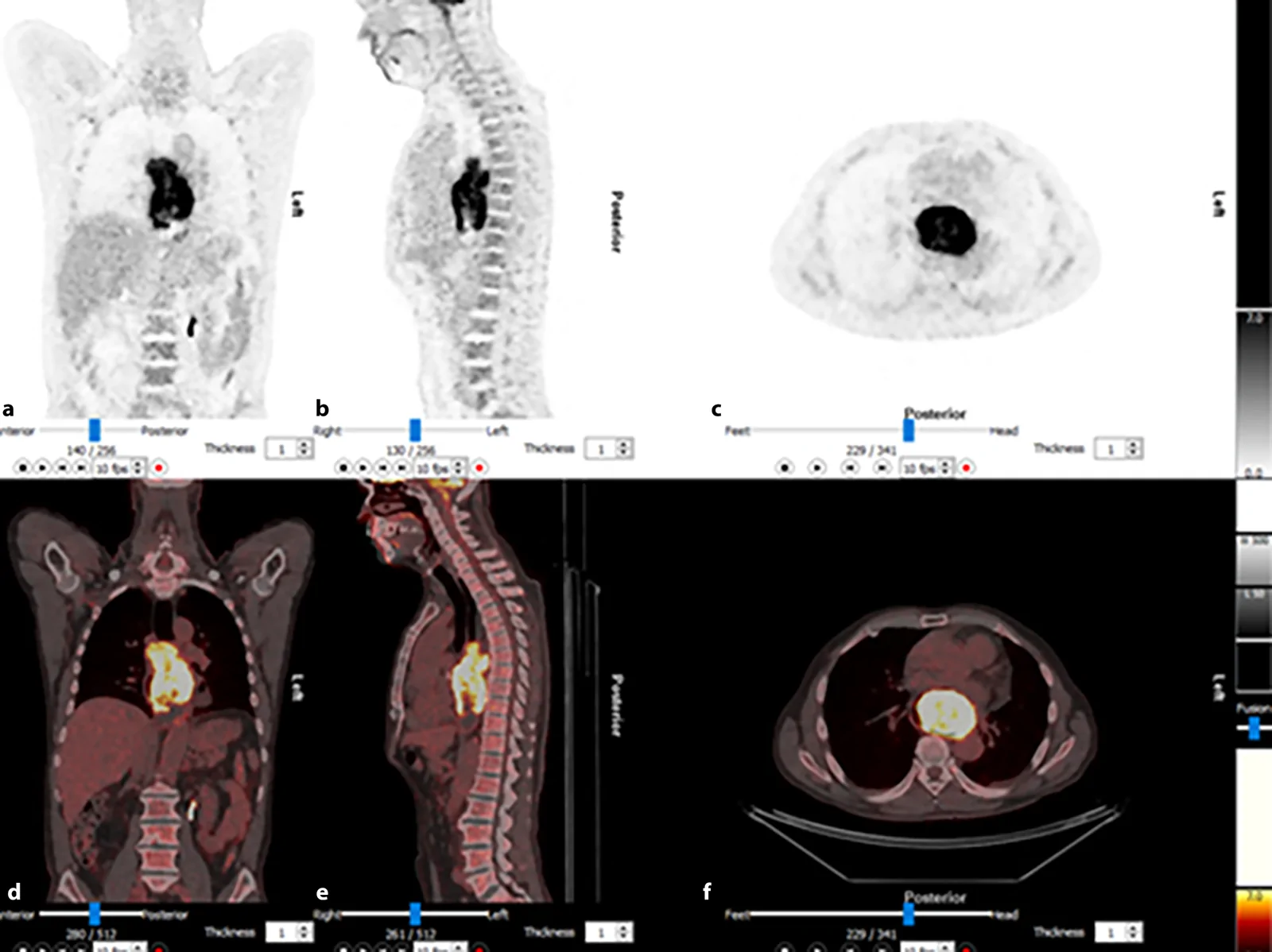
^18^F-fluorodeoxyglucose positron emission tomography/computed tomography (FDG-PET/CT) images of the thoracic region showing intense FDG uptake in the esophageal tumor. **a** Coronal PET/CT image demonstrating tracer uptake within the esophageal tumor. **b** Sagittal PET/CT image showing the tumor in relation to surrounding thoracic structures. **c** Axial PET/CT image confirming focal tracer accumulation within the lesion. **d** Coronal PET/CT image at a different level demonstrating the longitudinal extent of the tumor. **e** Sagittal PET/CT image at a different level showing the craniocaudal extent of the lesion. **f** Axial PET/CT image at a different level confirming persistent tracer uptake.

The corresponding ^18^F‑fluorodeoxyglucose positron emission tomography (FDG-PET) scan showed a considerable tracer uptake within the tumor with a maximum standardized uptake value (SUV_max_) of 15.6 highly suspicious of esophageal malignancy. There were no signs of lymph node involvement or distant metastases (shown in Fig. 3).

**Fig. 4:**
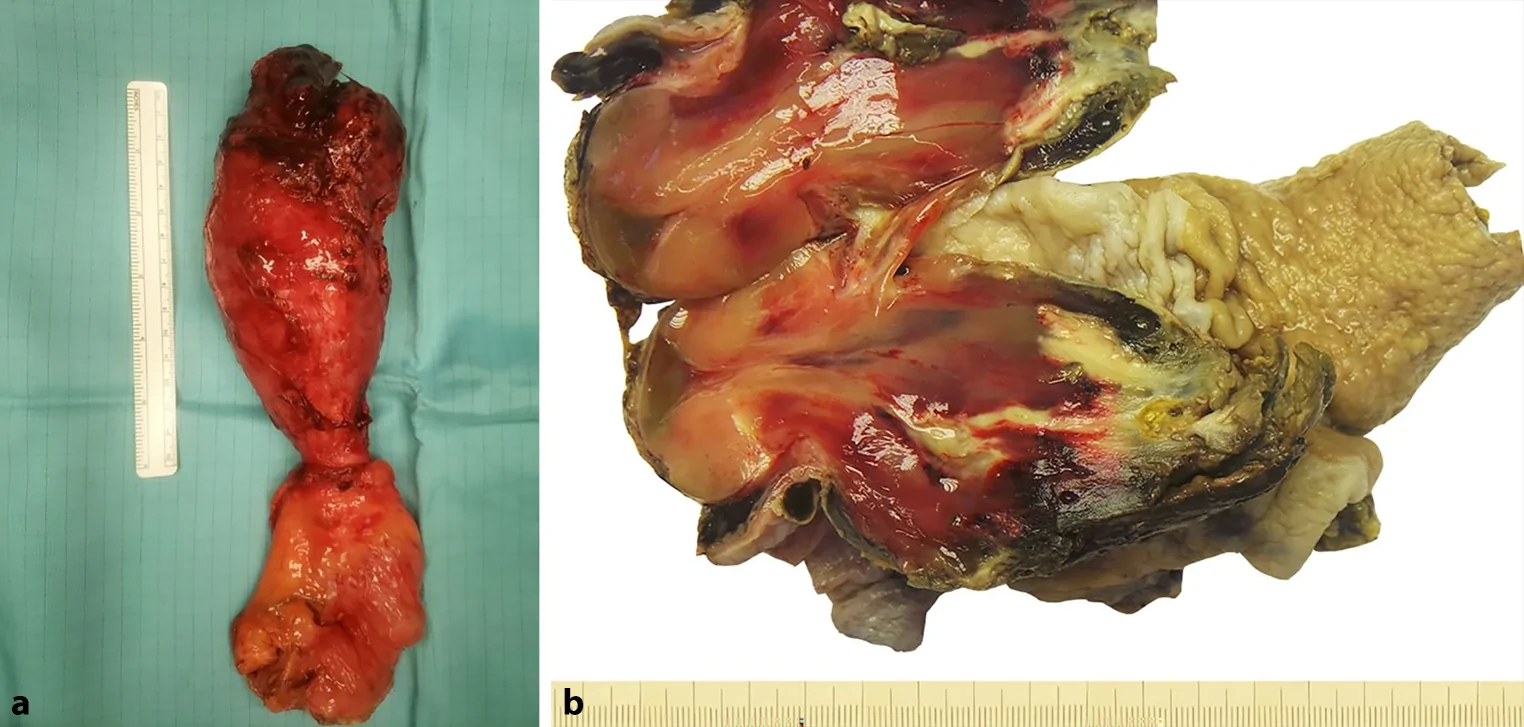
**a** Postoperative photograph of the completely resected esophagus containing the described plexiform fibromyxoma, **b** native photograph of the resected multinodular tumor with a tan glistering surface

**Fig. 5:**
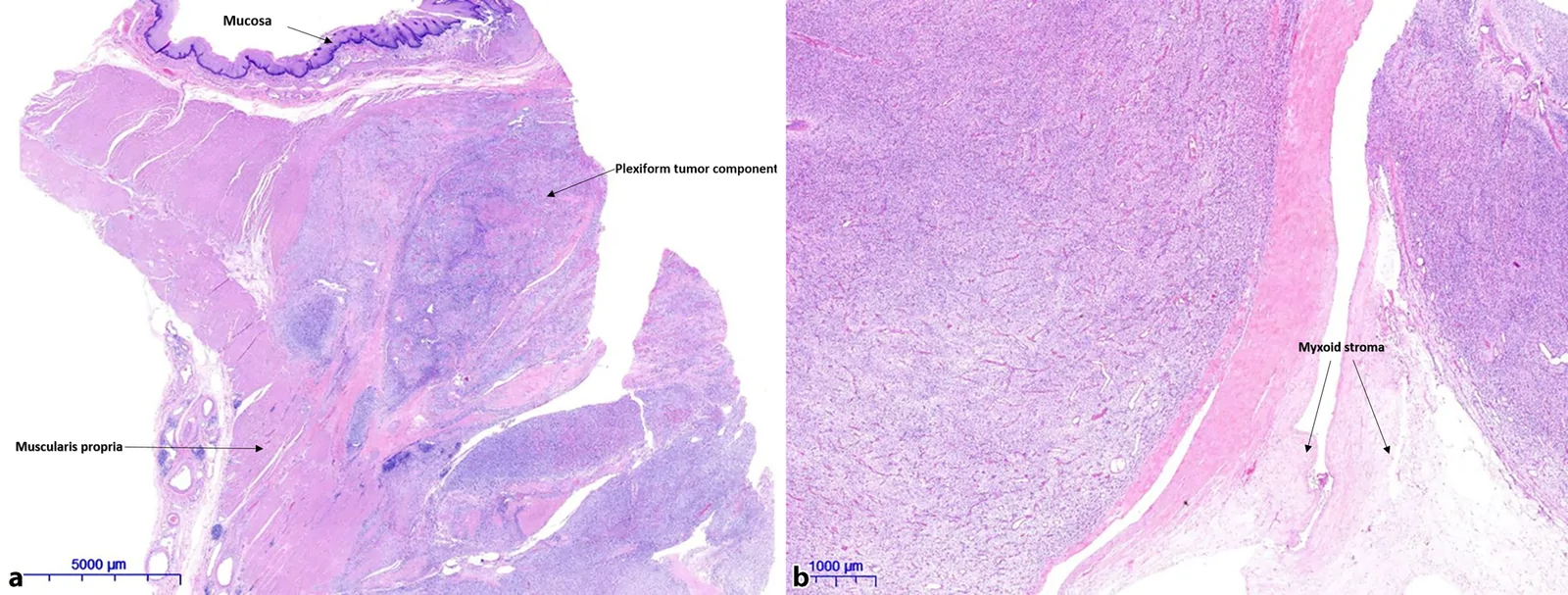
**a** Tumor shows a multinodular/plexiform growth pattern and infiltrates the esophageal wall layers (mucosa, submucosa and muscularis propria; arrows) (hematoxylin and eosin, scale bar = 5000 µm);. **b** Tumor composed of monomorphic spindle-shaped cells embedded in a loose extracellular matrix (hematoxylin and eosin, scale bar = 1000 µm).

In order to determine the correct treatment method, the findings were discussed within the weekly interdisciplinary tumor board. With respect to both the strong suspicion of malignancy in the absence of nodal and distant metastases and the exacerbated clinical symptoms reported by the patient, complete resection of the tumor was postulated as the treatment of choice. In the course of the detailed preoperative functional evaluation the cardiac ultrasound examination revealed extrinsic compression of the left atrium due to the tumor without impairment of cardiac function. According to the recommendation of the interdisciplinary tumor board transthoracic esophagectomy (shown in Fig. 4a) and reconstruction by gastric pull-up and cervical esophagogastrostomy were performed without complications. The postoperative course was uneventful, the patient fully recovered and was discharged for outpatient care 12 days after successful surgery. Grossly, the tumor was multinodular and showed a tan, glistering cut surface (shown in Fig. 4b). Histologically, the tumor was multinodular/plexiform in appearance and infiltrated the mucosa, submucosa, muscularis propria and fatty tissue (shown in Fig. 5a). It was composed of monomorphic tumor cells embedded in myxoid and collagenous stroma, with prominent network of small vessels (Fig. 5b). Immunohistochemistry showed negative staining for SMA, Desmin, Pankeratin, S100, CD34, ERG, MDM2, CDK4, CD117, DOG1, SOX10, MUC4, pan-TRK und HMB45. Molecular analysis using Archer Fusion Plex Pan Solid Tumor v2 (ArcherDX, Boulder, CO, USA) was performed and no gene rearrangement were detected including the absence of a MALAT1–GLI1 fusion.

## Discussion

Plexiform fibromyxoma is a very rare benign tumor of the upper gastrointestinal tract. In this context there is still little information in the literature about this special entity but it seems that the fundus and the antrum of the stomach are mostly affected; however, a predilection for gender or age has not been reported. The tumor-related clinical symptoms are determined by the size and the location of the tumor. Dysphagia affecting predominantly solid foods, weight loss and anemia have been described [1, 2]. Also, there is one case of rupture of the fibromyxoma and pyemia described in the literature [3]. Tumors of the antrum or fundus can develop without causing any clinical symptoms and are detected accidentally in the majority of the cases. About two thirds of the tumors are described to be associated with mucosal ulcerations with subsequent enhanced risk for severe bleeding and anemia [4].

In the current case the patient described dysphagia for solid food and weight loss for at least 2 months without consulting a doctor. In this context there are only two cases available in the literature. In the first case the tumor was described in the middle part of the esophagus of a 16-year-old teenager in 2014 with the size of 3.2 cm, the other one in a 56-year-old male patient, 6 × 3 cm in size [5, 6]. Both patients suffered from dysphagia representing the dominating clinical symptoms. Regarding the tumor size, the current case presents the largest fibromyxoma which has ever been described up to now.

In the present case the correct preoperative histological diagnosis could not be established, which is consistent with findings reported in the recent literature [7]. In our case an additional FDG-PET scan was performed to rule out possible malignancy. Due to concomitant severe ulcerous esophagitis the considerably high tracer uptake was mimicking a malignant esophageal tumor [8]. Endoscopy proved the inflammation of the tumor surface and the corresponding histological specimens confirmed chronic inflammatory exulceration without malignancy, which is in line with the recent literature [9]; however, the correct preoperative histological diagnosis of the plexiform fibromyxoma was not established even after several attempts of endoscopic biopsy and ultrasound guided fine needle aspiration. This is why the attempt of a diagnosis could be made for the first time during surgery. Surprisingly the results of the intraoperative frozen section led to the suspicion of an esophageal sarcoma due to the presence of cells with clear cytoplasm. Immunohistochemistry of the tumor showed a negative reaction with SMA, desmin, pankeratin, S100, CAM5.2, CD34, ERG, MDM2, CDK4, CD117, DOG1, SOX10, MUC4, pan-TRK and HMB45. The H3K27me3 expression is preserved with retained nuclear staining in tumor cells, beta-catenin showed unspecific dot-like cytoplasmic coloring and Ki-67 was positive in up to 25% of cells. No fusions were detected. The final diagnosis of the esophageal plexiform myxofibroma could only be established after careful histopathological examination.

**Fig. 6:**
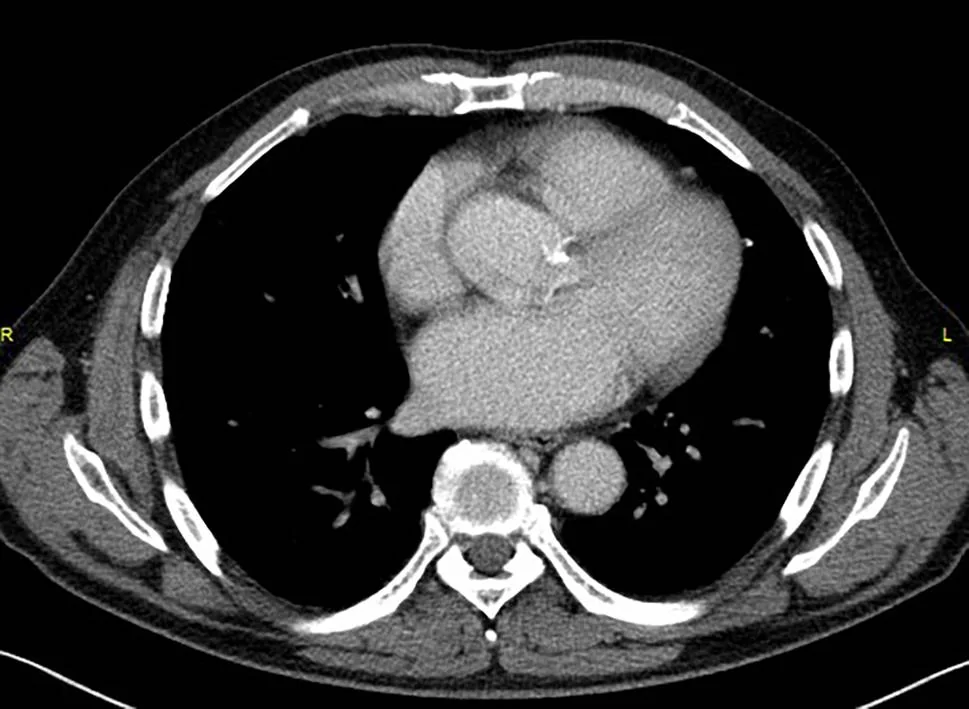
Computed tomography scan of the abdomen of the patient taken 27 months earlier: no sign of tumor in the distal esophagus

With respect to the course of disease, there is one aspect of the current case which has to be stressed: 27 months before the first endoscopic detection of the esophageal tumor a CT scan of the abdomen including the lower parts of the chest was performed, when the patient was suspected to suffer from diverticulitis. In this CT scan the distal part of the esophagus showed no evidence of tumor growth (shown in Fig. 6). With respect to this radiologic finding, we strongly suspect that in relation to this giant tumor size the presented esophageal plexiform fibromyxoma developed quickly within the last 2 years. This may be the reason for the quick development of the reported clinical symptoms, which had considerably deteriorated in the last months preceding surgery. After careful examination of the available literature there has been no comparable case reporting a similar speed of tumor growth.

Regarding this exceptional case plexiform fibromyxoma is a very rare benign tumor of the gastrointestinal system, especially in the esophagus. Dysphagia and unintended weight loss are the main symptoms. Preoperative diagnosis is relatively difficult, and rarely leading to a reliable result. It is essential to rule out gastrointestinal stromal tumors (GIST), as patients with advanced GIST may benefit from neoadjuvant treatment with imatinib [10]. The quick development of the tumor within 2 years shows the potential of a quick development of symptoms with rapid progress. This leads to a high pressure in treating these patients. The FDG-PET scan was highly positive in this patient. Despite an intensive search in PubMed there is no literature regarding positive or negative FDG-PET scan results. The patient also suffered from a serious esophagitis with ulceration and necrosis, which could be a reason for the FDG-PET positivity but as already mentioned, there were no PET scans described in former reports of plexiform fibromyxoma of the gastrointestinal system.

## Data Availability

The data underlying this article will be shared upon reasonable request to the corresponding author.
